# Improved Drill State Recognition during Milling Process Using Artificial Intelligence

**DOI:** 10.3390/s23010448

**Published:** 2023-01-01

**Authors:** Jarosław Kurek, Artur Krupa, Izabella Antoniuk, Arlan Akhmet, Ulan Abdiomar, Michał Bukowski, Karol Szymanowski

**Affiliations:** 1 Department of Artificial Intelligence, Institute of Information Technology, Warsaw University of Life Sciences, 02-776 Warsaw, Poland; 2 Department of Mechanical Processing of Wood, Institute of Wood Sciences and Furniture, Warsaw University of Life Sciences, 02-776 Warsaw, Poland

**Keywords:** tool state recognition, artificial intelligence, drill wear classification

## Abstract

In this article, an automated method for tool condition monitoring is presented. When producing items in large quantities, pointing out the exact time when the element needs to be exchanged is crucial. If performed too early, the operator gets rid of a good drill, also resulting in production downtime increase if this operation is repeated too often. On the other hand, continuing production with a worn tool might result in a poor-quality product and financial loss for the manufacturer. In the presented approach, drill wear is classified using three states representing decreasing quality: green, yellow and red. A series of signals were collected as training data for the classification algorithms. Measurements were saved in separate data sets with corresponding time windows. A total of ten methods were evaluated in terms of overall accuracy and the number of misclassification errors. Three solutions obtained an acceptable accuracy rate above 85%. Algorithms were able to assign states without the most undesirable red-green and green-red errors. The best results were achieved by the Extreme Gradient Boosting algorithm. This approach achieved an overall accuracy of 93.33%, and the only misclassification was the yellow sample assigned as green. The presented solution achieves good results and can be applied in industry applications related to tool condition monitoring.

## 1. Introduction

Automation is a key concept in industry, saving working time and increasing the task’s precision and overall repeatability. It is of great importance, especially in producing items in large quantities. Savings in working time are related to the efficiency of the plant, which can produce more in the same unit of time. The precision factor affects the production time [1] but also often increases the prestige due to the better quality of the final product. At the same time, repeatability is a challenge that ensures consistent production of the same products while maintaining a balance in working time and precision.

These three aspects together are important from the point of view of the furniture industry, discussed in [2,3]. The availability of a wide range of materials and complex production systems challenge the sustainability of production. A significant element that covers this industry is the drilling process. The materials used and the variability of parameters during work—including the structural diversity of wood and wood-based materials, mean that the level of precision may vary depending on many factors. Here, too, the continuity of production becomes important without sacrificing quality and increasing costs.

During the drilling process, due to various factors, such as mechanical, chemical or thermal processes occurring, the drill is blunting steadily. Those are important issues in machining science [4]. It is especially the case in materials such as melamine chipboard, where factors such as glue, the friction of wood or hard element contamination can influence this process [5,6,7]. Overall, tool quality, or in this case, drill bluntness, can greatly influence the quality of the final product [8].

Replacing the drill bit is a process that allows for reducing the damage to the material caused by the worn surface of the tool. Delaying replacement can result in poor production quality, including potential material costs. Replacing too early can also increase production costs by overinvesting in drill bits that do not necessarily need to be replaced [2]. The time required to exchange the drills is also a factor in that aspect, as during this process, the production is halted.

Currently, the entire tool exchange process is based on carefully calculating blade lifespan and the direct decision of the operator. Determining the optimal replacement time is also very costly, as current systems rely on estimated drill wear versus time and materials being drilled. For an automatic production system, manual verification of drill wear is unacceptable [9,10]. However, current prediction systems are inaccurate. It is, therefore, necessary to prepare a system that would allow for the correct classification of drill wear.

Various artificial intelligence solutions are widely used in the wood industry in general. Monitoring operational performance [11], optimizing drilling parameters [12] and AI algorithms perform well for various complex problems. The applications of such algorithms are also growing increasingly common in the wood industry in general, especially when it comes to problems encountered during the drilling process [13].

A similar analysis was performed, among others, by [14,15], incorporating an observational approach and the application of artificial intelligence together with the use of a Convolutional Neural Network (CNN). The implementation process required stopping the test system each time and taking pictures of the input and output holes. This was then used to determine the condition of the drill based on the effect of tearing the hole in the material. The method based on the graphic material (photo) ensured the effectiveness of determining the drill’s wear at nearly 80%. However, from the point of view of automation, the process requires an additional recorder that will carry out the data collection process and transfer it to a learned system recognizing the state, which will help make the right decision.

This article covers the process of developing the methodology adopted above based on the physical parameters of the drill system. The work focuses on verifying if it is possible to implement an algorithm that allows the optimization of the drill replacement process with automatic assessment using artificial intelligence.

The presented work focuses on a practical approach to tool state recognition that can be easily implemented. It achieves an accuracy level of over 90%. Feature generation in the form of STFT (Short-Time Fourier Transform) allows the extraction of more specific variables than standard FFT (Fast Fourier Transform) and has smaller time frames. Consequently, the frequency spectrum moves smoother over time, leading to higher accuracy. By testing 10 classifiers, the best one was selected, which can be easily implemented in practical, industrial applications.

## 2. Materials and Methods

### 2.1. Materials

The measuring station is a platform with mounted standard chipboard with dimensions of 300 × 150 (mm). Inside the board was drilled a 6 (mm) deep hole. The process uses a single-blade Faba head with a diameter of 40 (mm) with a replaceable fine-grained carbide blade. The spindle speed was 18,000 rpm with a feed per blade of 0.15 (mm).

While using the blade, there are three main states: hacking, stable state and error. Hacking is a short state directly after starting the tooling; the stable state will slowly decrease the overall tool state, while an error would result in a sudden decrease in product quality, rendering it unacceptable [16].

There are three different tool condition *states* determining the tool’s life—green, yellow and red. The Green state means new or unmarked tool (no initial data). The Red state denotes tool that requires replacement due to exceeded parameters ensuring effective operation. The intermediate state is yellow.

**Figure 1:**
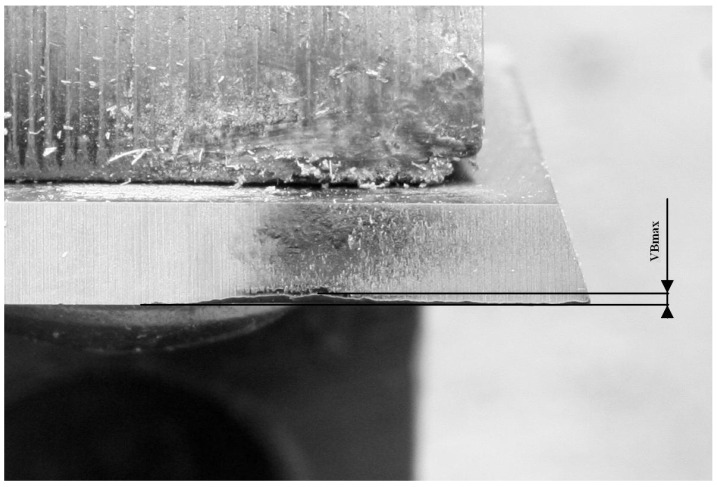
A microscopic photo of a drill bit wear.

For precise condition determination, the drill wear intervals were adopted as VBmax (Figure 1). The Green state was defined as the wear level between 0 and 0.15 (mm). The Yellow state is tool wear for the range between 0.151 and 0.299. The Red state is a range greater than 0.3.

During each of the experiments, tasks were temporarily interrupted, and the current condition of the blade was subjected to physical measurements using a Mitutoyo TM-505 microscope. It is well-suited for measuring dimensions and angles. Moreover, a Mitutoyo measuring microscope can be used to check the shape of screws and gears by attaching an optional reticle. Using this equipment, wear states have been measured and could be assigned to one of three wear states according to the following set of rules:

- if VBmax is in the range (0–0.15) mm, then it is a Green state—four different levels of wear state
- if VBmax is in the range (0.151–0.299) mm, then it is a Yellow state—two different levels of wear state
- if VBmax is in the range (>0.299) mm, then it is a Red state—two different levels of wear state

The experimental system has multiple sensors with the possibility of collecting 11 parameters, such as:

- force value in the (1) *X* and (2) *Y*-axes (Kistler 9601A sensor; Impexron GmbH, Pfullingen, Germany)
- (3) acoustic emission (Kistler 8152B sensor; Kistler Group, Winterthur, Switzerland)
- (4) noise level (Brüel & Kjær 4189 sensor; Brüel and Kjær, Nærum, Denmark)
- (5) vibration level (Kistler 5127B sensor; Kistler Group, Winterthur, Switzerland)
- (6) device-rated current (Finest HR 30 sensor; Micom Elektronika, Zagreb, Croatia)
- (7) device-rated voltage (Testec TT-Si9001 sensor; Testec, Dreieich, Germany)
- (8) head-rated current (Finest HR 30 sensor; Micom Elektronika, Zagreb, Croatia)
- (9) head-rated voltage (Testec TT-Si9001 sensor; Testec, Dreieich, Germany)
- (10) servo-rated current (Finest HR 30 sensor; Micom Elektronika, Zagreb, Croatia)
- (11) servo-rated voltage (Testec TT-Si9001 sensor; Testec, Dreieich, Germany)

National Instruments PCI-6111 measurement cards (for measuring acoustic emissions) and PCI-6034E (for measuring other parameters) were used for data acquisition of measurements from the sensors.

**Figure 2:**
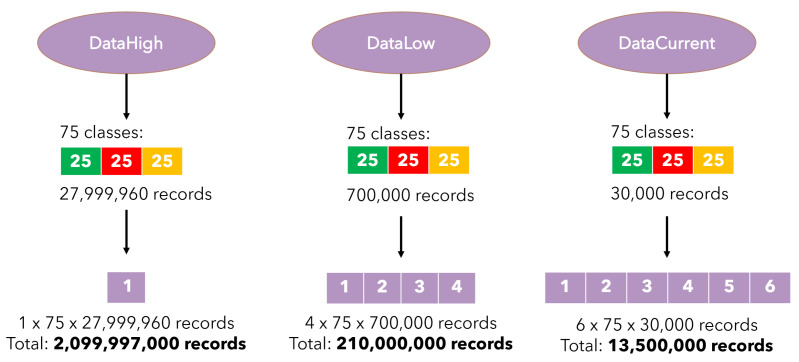
The structure of the data sets used in analysis.

All the collected research results were divided into three data sets (Figure 2): *DataHigh*, *DataLow* and *DataCurrent*. Each set included the three mentioned above states (25 files each) and a total number of data equal 225. The set of *DataHigh* included one parameter (Acoustic emission), each file with 27,999,960 records. The set *DataLow* consisted of four parameters (X/Y force value, noise level and vibration level), each file containing 700,000 records. The *DataCurrent* set included six parameters (current and voltage values for the device, head and motor drive), and the number of records was 30,000 per file (Table 1).

**Figure 3:**
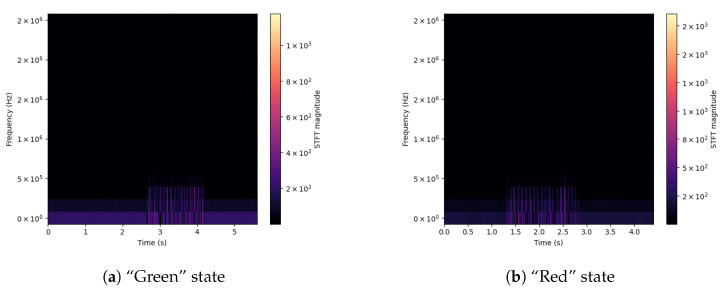
Signal spectrograms of Acoustic Emission for Green/Red states.

**Figure 4:**
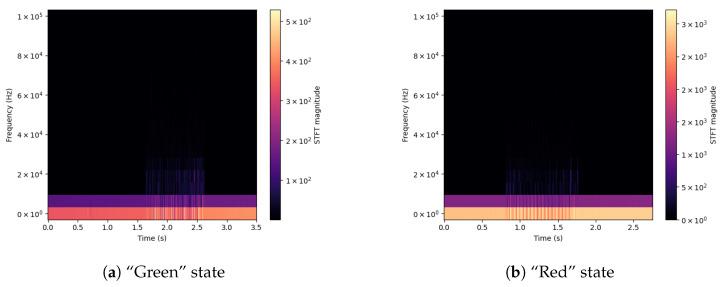
Signal spectrograms of Force X for Green/Red states.

**Figure 5:**
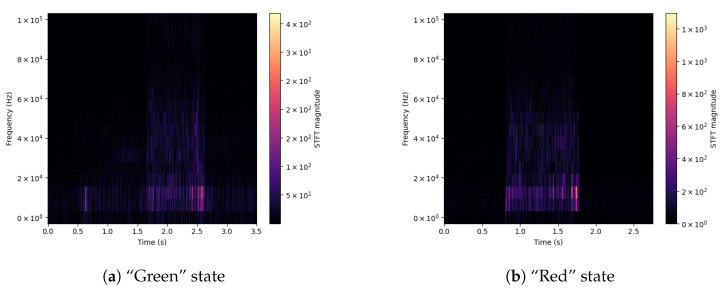
Signal spectrograms of Vibration for Green/Red states.

**Figure 6:**
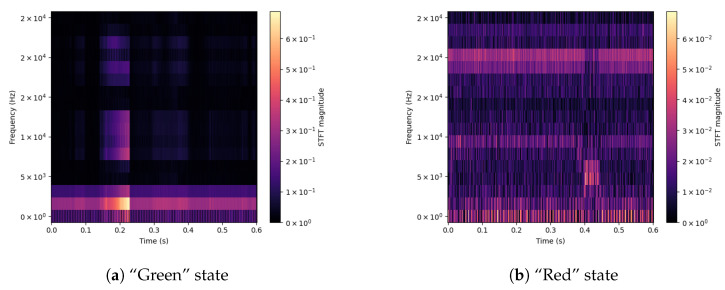
Signal spectrograms of DataCurrent for Green/Red states.

For a better understanding of the differences between signals, spectrograms were prepared for selected input parameters obtained from the used sensors. Extreme values of the drill state are shown—Green and Red. The shown signals concern the Acoustic Emission (Figure 3), applied Force on the X-axis (Figure 4), Vibration level (Figure 5) and Current consumption of the device (Figure 6).

### 2.2. Methods

**Figure 7:**
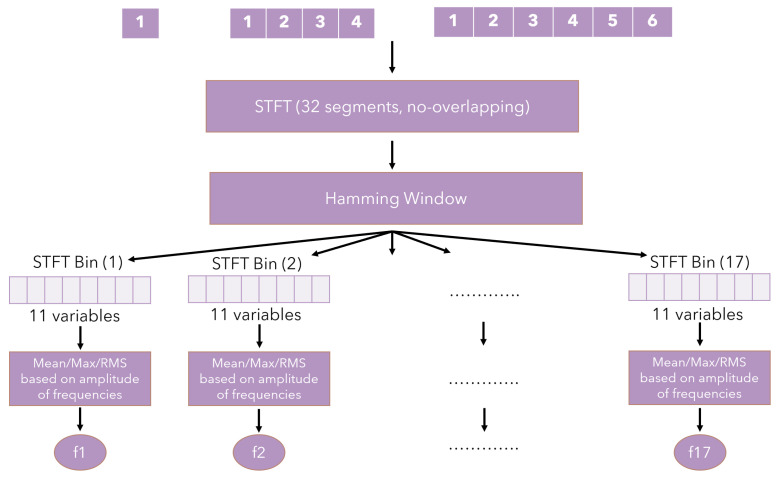
Feature generation flow chart.

In contrast to the optical methods of system analysis, the research carried out here allowed a collection of samples of the physical parameters of the machine. These data were imported and then processed (Figure 7) for further use in AI methods.

The first step was to perform a 32-segment Short-Time Fourier Transform (STFT) operation to split the samples by their frequency for all 11 input variables based on their sampling frequency (Table 1). To minimize data duplication, the transform did not include overlapping windows (the no overlap parameter was omitted). A Hamming window was used to define the range. Due to the symmetry of the system, only half of the bins (segments) were used for calculations, i.e., (32/2) + 1, which gives 17 bins in total.

For each of the obtained subsets of data, the mean value, maximum and effective value (RMS) was calculated. In the end, 51 variables (3×17) were obtained for each subset signal. Thus, 11(signals)×51 = 561 variables were obtained in the entire set.

**Figure 8:**
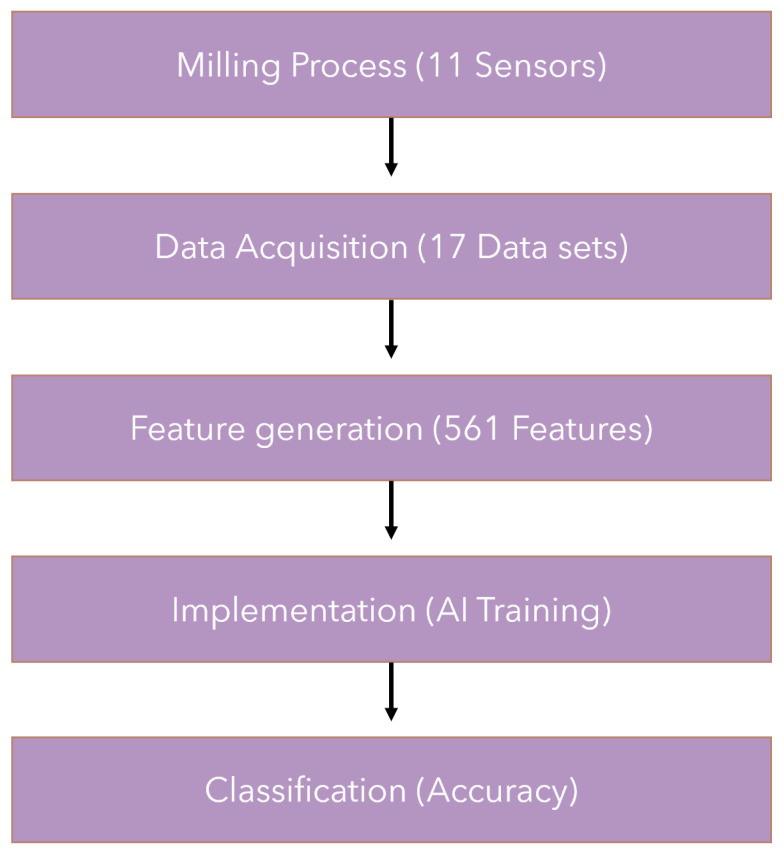
Key steps of the presented methodology.

For further effectiveness analysis, classification algorithms were prepared (Figure 8). The goal here was to evaluate a set with available previously prepared variables for which status it will qualify. The results were verified using ten popular classifiers: K-NN (K-Nearest Neighbors), GaussianNB (Gaussian Naive Bayes), MultinomialNB (Multinomial Naive Bayes), SGD (Stochastic Gradient Descent), DT (Decision Tree), RF (Random Forest), GB (Gradient Boosting), XGBoost (Extreme Gradient Boosting), LGBM (Light Gradient Boosting) and SVC (Support Vector Machine).

#### 2.2.1. K-Nearest Neighbors

The K-NN classifier is one of the most important non-parametric classification methods. In this method, the object being classified is assigned to the class to which most of its neighbors belong. In the case of an identical number of neighbors, the distances to each of them are calculated, and the smaller “distance” declares belonging [17,18].

The standard algorithm (based on Euclidean distance) k-NN is currently not often used. One of the approaches that improve the accuracy of the nearest neighbors classification is Neighborhood Components Analysis (NCA). The NCA algorithm maximizes a stochastic variant of the leave-one-out k-nearest neighbors scores on the training set.

NCA maximizes the sum over all samples *i* of the probability $p_{i}$ that *i* is correctly classified:

(1) $$\underset{L}{\arg\max}\sum_{i=0}^{N-1}p_{i}$$

(2) $$p_{i}=\sum_{j\in C_{i}}p_{ij}$$

(3) $$p_{ij}=\frac{\exp(-||Lx_{i}-Lx_{j}|{|}^{2})}{\sum_{k\neq i}\exp-(||Lx_{i}-Lx_{k}|{|}^{2})},\;p_{ii}=0$$

(4) $$||L\left(x_{i}-x_{j}\right){\left|\right|}^{2}={\left(x_{i}-x_{j}\right)}^{T}M\left(x_{i}-x_{j}\right)$$

(5) $$M=L^{T}L$$

where:

*N*—number of samples
$p_{i}$—probability of the sample being correctly classified
$C_{i}$—set of points in the same class as the sample
$p_{ij}$—softmax over Euclidean distances in the embedded space
$||L\left(x_{i}-x_{j}\right){\left|\right|}^{2}$—Mahalanobis distance metric

In the presented calculations, K-Nearest Neighbors had the following parameters:

- K = 5
- metrics = ‘minkowski’
- leaf_size = 30

#### 2.2.2. GaussianNB

The Naive Bayesian Classifier is based on Bayes’ theorem with the “naive” assumption of conditional independence between every pair of features given the value of the class variable.

The assumption of Bayes’ theorem is the following relationship [19,20]:

(6) $$P\left(y|x_{1},\ldots ,x_{n}\right)=\frac{P\left(y\right)P\left(x_{1},\ldots ,x_{n}|y\right)}{P(x_{1},\ldots ,x_{n})}$$

where *y* —class variable, $x_{i}$—dependent feature vector and under the naive conditional independence assumption:

(7) $$P\left(x_{i}|y,x_{1},\ldots ,x_{i-1},x_{i+1},\ldots ,x_{n}\right)=P\left(x_{i}|y\right)$$

and the assumption that the likelihood of the features is Gaussian:

(8) $$P\left(x_{i}|y\right)=\frac{1}{\sqrt{2\pi \sigma_{y}^{2}}}\exp\left(-\frac{{\left(x_{i}-\mu_{y}\right)}^{2}}{2\sigma_{y}^{2}}\right)$$

where the parameters $\sigma_{y}$ and $\mu_{y}$ are estimated using maximum likelihood.

GaussianNB did not require any parameters to be defined.

#### 2.2.3. MultinomialNB

The Multinomial Naive Bayesian Classifier is based on Bayes’ theorem also but multinomially distributed data [21,22]. The multinomial distribution is generated by vectors $\theta_{y}=\left(\theta_{y1},\ldots ,\theta_{yn}\right)$ for each class y, where n is the number of features and $\theta_{yi}$ is the probability $P(x_{i}|y)$ of feature i appearing in a sample belonging to class *y*.

Parameter $\theta_{y}$ is calculated by relative frequency counting:

(9) $${\hat{\theta }}_{yi}=\frac{N_{yi}+\alpha }{N_{y}+\alpha n}$$

where $N_{yi}=\sum_{x\in T}x_{i}$ is the number of times feature i appears in a sample of class y in the training set *T* and $N_{y}=\sum_{i=1}^{n}N_{yi}$ is the total count of all features for class *y*.

In the presented calculations, MultinomialNB did not require any parameters.

#### 2.2.4. Stochastic Gradient Descent

Stochastic Gradient Descent is an iterative method used to optimize the solution and its classification. It is based on the Robbins–Monro algorithm [23]. The goal is a stochastic approximation of the optimization of a given set (total gradient) by estimating it (randomly from a given subset). This solution is very computationally efficient for multidimensional problems but not very accurate in the convergence criterion [24,25].

The goal is to learn a linear scoring function $f\left(x\right)=w^{T}x+b$ with model parameters $w\in R^{m}$ and intercept b∈R and minimize the regularized training error, which is the following:

(10) $$E\left(w,b\right)=\frac{1}{n}\sum_{i=1}^{n}L\left(y_{i},f\left(x_{i}\right)\right)+\alpha R\left(w\right)$$

where:

*L*—loss function
*R*—regularization term that penalizes model complexity
α>0—is a non-negative hyperparameter that controls the regularization strength

The loss function is given by:

(11) $$L\left(y_{i},f\left(x_{i}\right)\right)=\max\left(0,1-y_{i}f\left(x_{i}\right)\right)$$

and the regularization term *R* is given by:

(12) $$R\left(w\right)=\frac{1}{2}\sum_{j=1}^{m}w_{j}^{2}={\left||w|\right|}_{2}^{2}$$

The core Stochastic Gradient Descent algorithm is an optimization method for unconstrained optimization problems. SGD approximates the true gradient of E(w,b) by considering a single training example at a time. The algorithm iterates over the training examples and, for each example, updates the model parameters according to the update of the following rule:

(13) $$w\leftarrow w-\eta \left[\alpha \frac{\partial R(w)}{\partial w}+\frac{\partial L(w^{T}x_{i}+b,y_{i})}{\partial w}\right]$$

where:

η—learning rate
*b*—intercept

In the presented calculations, Stochastic Gradient Descent had the following parameters:

- *L*—Hinge loss function
- max_iter = 1000
- validation fraction = 10%
- α = 0.0001
- penalty = L2

#### 2.2.5. Decision Tree

Decision Tree is the simplest and most popular classifier based on scenarios of decision criteria [26]. Narrowing down the results by range classes is the basis for decision-making. The algorithm’s performance for smaller training sets may lead to erroneous results [27].

Decision Tree recursively partitions the feature space in the way that samples with the same labels are grouped together.

We assume that:

$x_{i}\in R^{n}$—training vectors
$y\in R^{l}$—label vector
*m*—number of node
$Q_{m}$—data at node *m*
$n_{m}$—number of samples at node *m*

Then for each candidate split $\theta =(j,t_{m})$ consisting of a feature *j* and threshold $t_{m}$, we split the data into $Q_{m}^{left}\left(\theta \right)$ and $Q_{m}^{right}\left(\theta \right)$ subsets.

(14) $$\begin{array}{c} Q_{m}^{left}\left(\theta \right)=\left\{\left(x,y\right)|x_{j}\leq t_{m}\right\}\\ Q_{m}^{right}\left(\theta \right)=Q_{m}\setminus Q_{m}^{left}\left(\theta \right) \end{array}$$

The decision of which node should be split is made by the following rule:

(15) $$G\left(Q_{m},\theta \right)=\frac{n_{m}^{left}}{n_{m}}H\left(Q_{m}^{left}\left(\theta \right)\right)+\frac{n_{m}^{right}}{n_{m}}H\left(Q_{m}^{right}\left(\theta \right)\right)$$

where H()—loss function, very often as Gini:

(16) $$H\left(Q_{m}\right)=\sum_{k}p_{mk}\left(1-p_{mk}\right)$$

(17) $$p_{mk}=\frac{1}{n_{m}}\sum_{y\in Q_{m}}I\left(y=k\right)$$

where:

*k*—Number of classes
*m*—Number of nodes

It is then recursively computed for subsets $Q_{m}^{left}\left(\theta^{*}\right)$ until the maximum allowable depth is reached, $n_{m}<\min_{samples}$ or $n_{m}=1$.

In the presented calculations, Decision Tree had the following parameters:

- min_samples_leaf = 1
- loss function = Gini

#### 2.2.6. Random Forest

Random Forest is an example of an algorithm that uses ensemble methods. The idea of ensemble methods is to combine the predictions of several base classifiers built with a given learning algorithm in order to improve the robustness in comparison to a single estimator [28].

In Random Forest, each tree in the ensemble is built from a sample drawn with replacement (subset random samples) from the training set. During the splitting of each node, the best split is found from a random subset of features [29,30].

Individual Decision Tree classifier has high variance and tends to overfit. Thanks to the randomness approach, the variance of the Random Forest classifier decreases. Moreover, the injected randomness decoupled prediction errors, and by the average approach of those predictions, some errors can cancel out. The variance reduction often tends to an overall better model.

In the presented calculations, Random Forest had the following parameters:

- min_samples_leaf = 1
- loss function = Gini
- base classifier = Decision Tree

#### 2.2.7. Gradient Boosting

The next algorithm is Gradient Boosting, a method that uses dependencies in the previous steps of the result prediction. After each iteration, the result of the predictor is corrected for the residuals from the training set, and a new predictor is created, devoid of the error of the previous iteration. The algorithm was first described in [31,32] and is the starting point for many other much-improved methods [33,34].

Gradient Boosting, Gradient Tree Boosting or Gradient Boosted Decision Trees (GBDT) is a generalization of boosting to arbitrary differentiable loss functions.

Gradient Boosting for classification is based on a regression approach, but the output cannot be a class since the trees predict continuous values, so the appropriate mapping should be applied.

Gradient Boosting is an additive model where prediction ${\hat{y}}_{i}$ for a given features $x_{i}$ is based on the following rule:

(18) $${\hat{y}}_{i}=F_{M}\left(x_{i}\right)=\sum_{m=1}^{M}h_{m}\left(x_{i}\right)$$

where:

$h_{m}$—weak learners
*M*—number of weak learners

Gradient Boosting is a greedy algorithm:

(19) $$F_{m}\left(x\right)=F_{m-1}\left(x\right)+h_{m}\left(x\right)$$

where $h_{m}$ minimize a sum of losses $L_{m}$ from the previous ensemble $F_{m-1}$:

(20) $$h_{m}=\arg\min_{h}L_{m}=\arg\min_{h}\sum_{i=1}^{n}l\left(y_{i},F_{m-1}\left(x_{i}\right)+h\left(x_{i}\right)\right),$$

where $l(y_{i},F\left(x_{i}\right))$—loss function

The mapping from the value $F_{M}\left(x_{i}\right)$ to a class is loss-dependent. For the log-loss, the probability that $x_{i}$ belongs to the positive class is based on the following rule:

(21) $$p\left(y_{i}=1|x_{i}\right)=\sigma \left(F_{M}\left(x_{i}\right)\right)$$

where σ—sigmoid function

In the case of multiclass classification, K trees (K classes) are built at each of the *M* iterations. The probability that $x_{i}$ belongs to class k is calculated using softmax of the $F_{M,k}\left(x_{i}\right)$ values.

In the presented calculations, Gradient Boosting had the following parameters:

- *M* = 100
- loss function = ‘log-loss’
- max_depth = 3
- learning_rate = 0.1
- min_samples_leaf = 1

#### 2.2.8. Extreme Gradient Boosting

Extreme Gradient Boosting (XGBoost) is an improved version of the classic solution based on Gradient Boosting [32,34,35,36].

XGBoost has many advantages in comparison to standard Gradient Boosting [37]:

- regularization rules,
- parallel processing,
- an in-built feature to handle missing values,
- built-in cross-validation technique,
- tree pruning feature.

In the presented calculations, Extreme Gradient Boosting had the following parameters:

- *M* = 100
- loss function = ‘log-loss’
- max_depth = 3
- learning_rate = 0.1
- min_samples_leaf = 1

#### 2.2.9. Light Gradient Boosting

Another algorithm that uses Gradient Boosting is LGBM. Unlike algorithms based on random trees, such as XGBoost, it does not rely on sorting to find the best split point. It is based on Decision Trees using the decision histogram, which provides the possibility to follow the path of the expected least loss in time [38,39].

In comparison to XGBoost, LGBM has vertical growth (leaf-wise) that results in more loss reduction, and it tends to a higher accuracy, while XGBoost has horizontal growth (level-wise).

In the presented calculations, Light Gradient Boosting had the following parameters:

- *M* = 100
- loss function = ‘log-loss’
- learning_rate = 0.1
- reg_alpha = 0
- reg_lambda = 0
- boosting_type = ‘gbdt’

#### 2.2.10. Support Vector Machine

The Support Vector Machine is the learning method for classification [40,41] based on correctly mapping data to multidimensional space and applying a function separating these data, declaring decision classes. A Support Vector Machine builds a hyperplane or set of hyperplanes in a high-dimensional space based on kernel functions. The goal is to maximize the separation margin. The separation margin is the largest distance to the nearest training data points of any class (support vectors) [42].

The main idea is to maximize the margin (by minimizing ${\left||w|\right|}^{2}=w^{T}w$) and penalize the margin when a sample is misclassified:

(22) $$\begin{array}{c} \min_{w,b,\zeta }\frac{1}{2}w^{T}w+C\sum_{i=1}^{n}\zeta_{i}\\ \begin{array}{cc} \mathrm{subject}\mathrm{to} & y_{i}\left(w^{T}\phi \left(x_{i}\right)+b\right)\geq 1-\zeta_{i},\\ \; & \zeta_{i}\geq 0,i=1,...,n \end{array} \end{array}$$

where:

*C*—penalty term that controls the penalty strength
$\zeta_{i}$—distance samples from their correct margin boundary

The main problem can be changed to a dual problem:

(23) $$\begin{array}{c} \min_{\alpha }\frac{1}{2}\alpha^{T}Q\alpha -e^{T}\alpha \\ \begin{array}{cc} \mathrm{subject}\mathrm{to} & y^{T}\alpha =0\\ \; & 0\leq \alpha_{i}\leq C,i=1,...,n \end{array} \end{array}$$

where:

$\alpha_{i}$—dual coefficients
*e* is the vector of all single coefficients. The positive semidefinite matrix is $Q_{ij}=y_{i}y_{j}K\left(x_{i},x_{j}\right)$ and $K\left(x_{i},x_{j}\right)=\phi {\left(x_{i}\right)}^{T}\phi \left(x_{j}\right)$ is the kernel.

In the case of multi-class classification, the “one-versus-one” approach is often applied, which means that m*(m − 1)/2 classifiers are constructed where m is the number of classes.

In the presented calculations, SVM had the following parameters:

- *C* = 30,000
- kernel = ‘RBF’
- gamma = 1/561

#### 2.2.11. General Implementation

The entire implementation was prepared in Python programming language (version 3.9.9) with PyCharm editor enabled (version 2022.2.3 Professional Edition). PyCharm is an integrated development environment (IDE) widely used for development. It provides functionalities such as: code analysis, graphical debugger, integrated unit tester, integration with version control systems (such as Git), etc. PyCharm is developed by the Czech company JetBrains.

Additionally, scikit-learn—one of the widely used libraries, was used (open-source data analytics library). It is the gold standard for machine learning (ML) in the Python ecosystem. This library has been applied for data preprocessing, pipeline, model selection, classifiers implementation, hyperparameters optimization, building classification reports, confusion matrices, etc.

Pycharm has been installed on a Windows 10 system and managed the whole Python project, but python code has been executed remotely (via ssh) on an Ubuntu 18.04.6 LTS (Bionic Beaver) machine that is dedicated to machine learning and deep learning projects.

All experiments were performed on hardware (Ubuntu operating system) with the following specifications:

- Processor: AMD RYZEN THREADRIPPER 2990WX (32C 64T) 4.3 GHz
- Motherboard: AsRock X399 TAICHI
- Memory: 8 × ADATA XPG SPECTRIX DDR4 16 GB D41 3000MHz (128 GB RAM)
- Graphics Card: 2 × Nvidia GeForce RTX Titan 24GB GDDR6 (48 GB RAM)
- Drive SSD: 2 × WD BLACK 1TB WDS100T3X0C1TB (PCIE)
- Drive HDD: 1 × WD RED PRO 8TB WD8003FFBX 3.5” (SATA)
- Power Supply: BE QUIET! DARK POWER PRO 11 1000 W
- Cooling: BE QUIET! Silent Loop BW003 280 mm
- Network: 10GbE SFP+

## 3. Discussion

In the conducted research, the ten previously mentioned classification algorithms were used in the cross-validation method on the input sets of the collected data. Each set of data (*DataHigh*, *DataLow*, *DataCurrent*) for each state (“Green”, “Yellow” and “Red”) was prepared using a Pareto rule (also called *80/20*), which was implemented learning on 80% of the data set and testing was performed on the remaining 20%.

The spectrograms of the selected time courses presented in Figure 3, Figure 4, Figure 5 and Figure 6 show clear changes in the tendency of signals, which, however, can be correctly interpreted. A face-to-face comparison of the extreme states shows how the signal changes with each drilling job for a drill marked as new or excellent (“Green”) and one that is worn (“Red”).

In the case of Acoustic Emission (*DataHigh*, Figure 3) for state “Green” (a), slight changes in the value of the frequency level can be noticed in the final stage of the task. For a drill marked with state “Red” (b), the emission level in the analyzed frequency range is distributed over the entire time interval of the task stage, which may mean that the source of interference is in the indicated drill.

For the spectrogram of the applied Force in the *X*-axis (*DataLow*, Figure 4), both in the case of a drill with the state “Green” (a) and “Red” (b), it is uniform throughout the task. However, there is a visible change in the force value, which increased six times in the case of a worn drill bit.

The system’s vibration level (*DataLow*, Figure 5) corresponds to the mentioned acoustic emission—for the “Green” drill (a), the vibration level is not only low but also slightly increases at the end of the stage works. For a worn drill bit (b), the vibration level is much higher (up to four times) and is present throughout the task.

The last spectrogram comparison set of all 11 features available is the current consumption value (*DataCurrent*, Figure 6) of the device during the task. According to the principle of operation of the electrical device, the current consumption increases only for the system that starts operation, and in the case of achieving full stabilization, the value drops at the very end of the task. This can be seen from the spectrogram for state “Green” (a). In the case of a worn (b) drill, the value of the current consumption varies and can be read as unstable operation of the device.

As a result of the analysis, summaries were developed for each classification method, including the precision of classifying the state to its actual class in relation to the assumptions. Each misclassification against the expected value resulted in a lower prediction level for a single state (e.g., expected “Red” has been classified as “Green”) and the entire set (e.g., how many times “Red” is classified as “non-Red”). The results of individual classifications, along with the accuracy of classification for a given algorithm, are presented in Table 2, Table 3, Table 4, Table 5, Table 6, Table 7, Table 8, Table 9, Table 10 and Table 11.

The first of the considered parameters for the solution quality is the overall algorithm accuracy. Out of the total results (Table 12), XGBoost (*Extreme Gradient Boosting*) is the classifier with the highest prediction score, 93.33%. Two more classifiers: GB (*Gradient Boosting*) and DT (*Decision Tree*), achieved a result of 86.66%. The remaining methods did not exceed the threshold of 85%, which can be considered a poor result.

**Figure 9:**
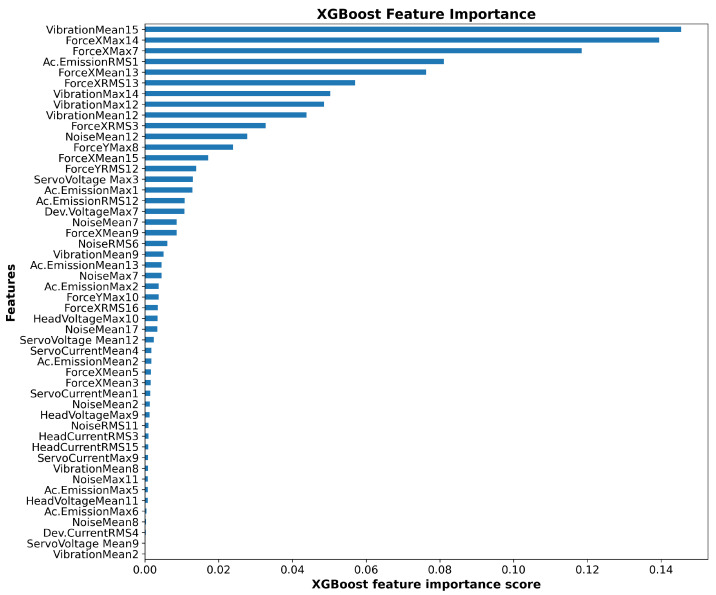
XGBoost feature importance for all (50) important features.

Algorithm XGBoost chose only 50 features to build the model. The ranking features are depicted in Figure 9. The number at the end of the feature name is regarding the number of the segment in the STFT algorithm (maximum 17).

On the basis of Table 13, we can see that the main signals that XGBoost used to build the model are Force X, Noise, Ac. Emission and Vibration.

**Figure 10:**
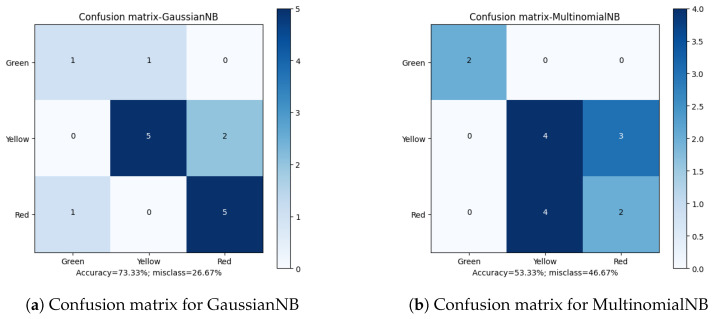
Confusion matrices for GaussianNB and MultinomialNB classifiers.

**Figure 11:**
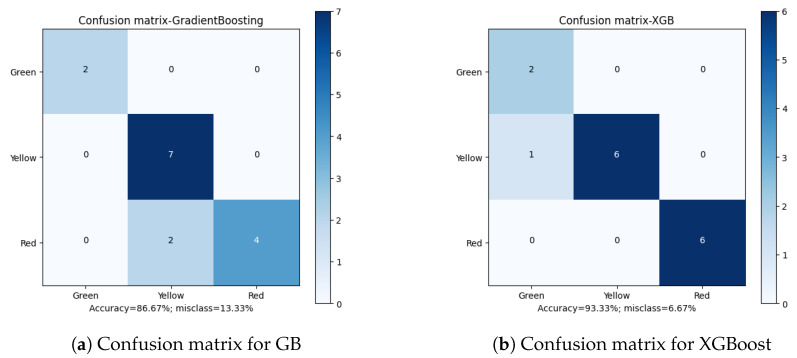
Confusion matrices for Gradient Boosting and Extreme Gradient Boosting classifiers.

**Figure 12:**
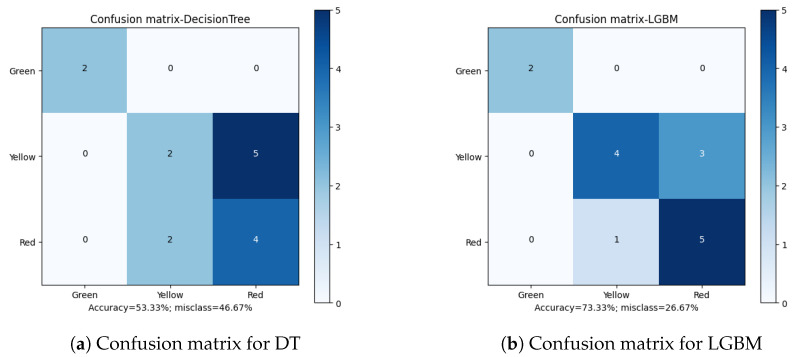
Confusion matrices for Decision Tree and Light Gradient Boosting classifiers.

**Figure 13:**
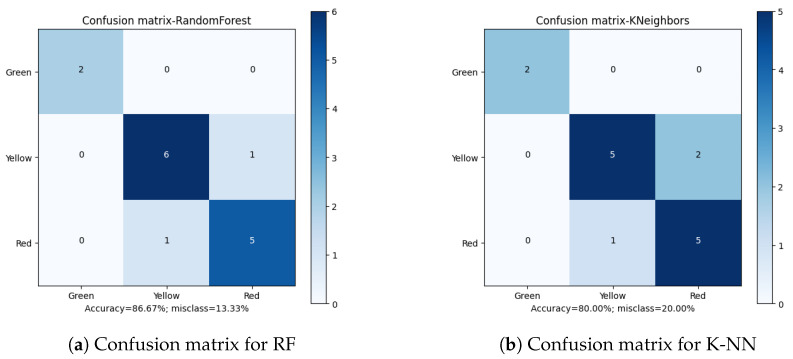
Confusion matrices for Random Forest and K-Nearest Neighbors classifiers.

**Figure 14:**
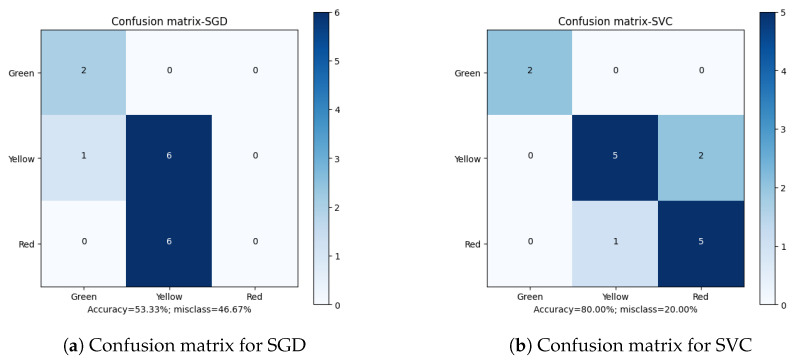
Confusion matrices for Stochastic Gradient Descent and Support Vector Machine classifiers.

Apart from the overall accuracy, the misclassification rate is another important factor in defining the quality of the final results. Analyzing the Confusion Matrices (Figure 10, Figure 11, Figure 12, Figure 13 and Figure 14), it can be clearly stated that in the case of the XGBoost classifier, there were FP cases (False Positive) for state “Yellow”, which defined the wear of the drill as good (“Green”). However, it was marked as already worn. Taking into account the degree of precision, the error is significant, but it still does not declassify the algorithm as suitable for the adopted methodology. This level of accuracy allows the method to be put into practice with certainty. In the case of algorithms that obtained second and third place in terms of classification accuracy (achieving the same score), there were cases of incorrect cross-classification. The “Yellow” state was assigned to the “Red” drills and vice versa. Such scenarios can result in real losses in the case of this type of error.

What is most important is the lack of “Green-Red” and “Red-Green” errors in the best-performing algorithms. From the tool wear and overall production process point of view, such misclassifications have the most impact on the solution quality. In the presented cases, no such errors occurred during method evaluation. Additionally, in the case of the XGBoost algorithm, both border states (“Green” and “Red”) had 100% accuracy when being assigned, with the only errors occurring in the “Yellow” state. This minimizes the two main risks: first, the tool being exchanged too early (or too often), increasing the production downtime; secondly, the tool being in unsatisfactory condition to be continuously integrated in the production process, resulting in poor quality products and loss for the manufacturer.

Overall, the misclassification rate, when combined with high accuracy obtained by the best methods, provided a viable solution that is applicable to the presented task.

## 4. Conclusions

The article presents a new approach to the problem of drill wear classification. The tests were not based on a visual representation of the holes but on measurements of the drilling system in terms of physical parameters, such as noise levels, current/voltage values and vibrations. Each of the measurements was saved in a separate data set, the values of which were parameterized in the time window corresponding to the changes in the real research interval.

The presented algorithms determined the level of accuracy in this particular study. Of the available classification solutions, three achieved a precision score above 85%. This value is satisfactory from the industry’s point of view, but it may be too low to minimize the damage and costs of drilling holes in wood and wood-based materials in this case. The XGBoost classification algorithm was the only one to achieve a precision value above 93% on all 17 time windows and test sets. Such a result indicates that this methodology can determine the practical implementation in production. The XGBoost algorithm was also very accurate in terms of misclassification errors, as the only state that was not correctly assigned was the “Yellow” one. The lack of “Red-Green” and “Green-Red” errors is an additional advantage of the presented method.

The proposed solution has a very high level of certainty, but it is still possible to improve and re-verify in the case of an analysis based on real, more extensive sets of data. Such information may come from furniture factories or precision wood processing plants, increasing the diversity and overall representation of the processing and environmental properties.

## Figures and Tables

**Table 1:** The structure of the data variables in data sets.

Data Set	Variable	Length of 1 Trial	Sampling Frequency (Hz)	Measure Time (s)
DataHigh	Ac. Emission	27,999,960	5,000,000	5.59
DataLow	Force X	700,000	200,000	3.50
DataLow	Force Y	700,000	200,000	3.50
DataLow	Noise	700,000	200,000	3.50
DataLow	Vibration	700,000	200,000	3.50
DataCurrent	Dev. Current	30,000	50,000	0.60
DataCurrent	Dev. Voltage	30,000	50,000	0.60
DataCurrent	Head Current	30,000	50,000	0.60
DataCurrent	Head Voltage	30,000	50,000	0.60
DataCurrent	Servo Current	30,000	50,000	0.60
DataCurrent	Servo Voltage	30,000	50,000	0.60

**Table 2:** Classification report for the GaussianNB classifier.

Class	Precision	Recall	F1-Score
Green	0.50	0.50	0.50
Yellow	0.83	0.71	0.77
Red	0.71	0.83	0.77
Accuracy			**0.73 **

**Table 3:** Classification report for the MultinomialNB classifier.

Class	Precision	Recall	F1-Score
Green	1.00	1.00	1.00
Yellow	0.50	0.57	0.53
Red	0.40	0.33	0.36
Accuracy			**0.53 **

**Table 4:** Classification report for the GB classifier.

Class	Precision	Recall	F1-Score
Green	1.00	1.00	1.00
Yellow	0.78	1.00	0.88
Red	1.00	0.67	0.80
Accuracy			**0.87 **

**Table 5:** Classification report for the XGBoost classifier.

Class	Precision	Recall	F1-Score
Green	0.67	1.00	0.80
Yellow	1.00	0.86	0.92
Red	1.00	1.00	1.00
Accuracy			**0.93 **

**Table 6:** Classification report for the DT classifier.

Class	Precision	Recall	F1-Score
Green	1.00	1.00	1.00
Yellow	0.50	0.29	0.36
Red	0.44	0.67	0.53
Accuracy			**0.53 **

**Table 7:** Classification report for the LGBM classifier.

Class	Precision	Recall	F1-Score
Green	1.00	1.00	1.00
Yellow	0.80	0.57	0.67
Red	0.62	0.83	0.71
Accuracy			**0.73 **

**Table 8:** Classification report for the RF classifier.

Class	Precision	Recall	F1-Score
Green	1.00	1.00	1.00
Yellow	0.86	0.86	0.86
Red	0.83	0.83	0.83
Accuracy			**0.87 **

**Table 9:** Classification report for the K-NN classifier.

Class	Precision	Recall	F1-Score
Green	1.00	1.00	1.00
Yellow	0.83	0.71	0.77
Red	0.71	0.83	0.77
Accuracy			**0.80 **

**Table 10:** Classification report for the SGD classifier.

Class	Precision	Recall	F1-Score
Green	0.67	1.00	0.80
Yellow	0.50	0.86	0.63
Red	0.00	0.00	0.00
Accuracy			**0.53 **

**Table 11:** Classification report for the SVC classifier.

Class	Precision	Recall	F1-Score
Green	1.00	1.00	1.00
Yellow	0.83	0.71	0.77
Red	0.71	0.83	0.77
Accuracy			**0.80 **

**Table 12:** Classification report for all classifiers.

Model	Parameters	Accuracy (%)
GaussianNB	var_smoothing = 1 $\times \;10^{-9}$	73.33
MultinomialNB	alpha = 1.0	53.33
Gradient Boosting	learning_rate = 0.1, n_estimators = 100	86.66
**Extreme Gradient Boosting **	learning_rate = 0.1, n_estimators = 100	**93.33**
Decision Tree	min_samples_split = 2, min_samples_leaf = 1	53.33
Light Gradient Boosting	num_leaves = 31, learning_rate = 0.1, n_estimators = 100	73.33
Random Forest	n_estimators = 100, min_samples_split = 2, min_samples_leaf = 1	86.66
K-Nearest Neighbors	n_neighbors = 5	80.00
Stochastic Gradient Descent	alpha = 0.0001, epsilon = 0.1	53.33
Support Vector Machine	C = 1.0, kernel = RBF, gamma = 0.1	80.00

**Table 13:** Ranking list of the number of signal occurrences in XGBoost features.

Name of Signal	Number of Signal Occurrences in XGBoost Features
ForceX	10
Noise	9
Ac.Emission	8
Vibration	7
ForceY	3
HeadVoltage	3
ServoCurrent	3
ServoVoltage	3
HeadCurrent	2
Dev.Current	1
Dev.Voltage	1

## Data Availability

Not applicable.
